# Intraneural injection of corticosteroids to treat nerve damage in leprosy: a case report and review of literature

**DOI:** 10.1186/1752-1947-2-381

**Published:** 2008-12-09

**Authors:** Sherine G Nashed, Tarek A Rageh, Emad S Attallah-Wasif, Alaa A Abd-Elsayed

**Affiliations:** 1 Assiut Dermatology and Leprosy Clinic, Assiut, Egypt; 2 Neurology Department, Assiut University Hospital, Assiut, Egypt; 3 Rush University Medical Center, Chicago, IL, USA; 4 Public Health and Community Medicine Department, Faculty of Medicine, Assiut University, Assiut, Egypt

## Abstract

**Introduction:**

Nerve damage in leprosy patients leads to deformities and disabilities. Oral corticosteroids are given early to prevent permanent injury. We present a new approach to treat well-established nerve damage with local injection of corticosteroids.

**Case presentation:**

A 60-year-old leprosy patient presented with right claw hand deformity secondary to right ulnar and median neuropathy. Monthly intraneural injection of corticosteroids resulted in improvement in sensory and motor function of his right hand over a 6-month period. Nerve conduction velocity testing documented the success of our therapy.

**Conclusion:**

We report the first case of successful nerve regeneration in neglected neuropathy secondary to leprosy after local injection of corticosteroids. Intraneural extra-fascicular injection of corticosteroids improved the sensory and motor nerve function in our patient with borderline leprosy regardless of the duration of nerve function loss.

## Introduction

Leprosy is a chronic granulomatous disease caused by *Mycobacterium leprae*. It has almost been eliminated from developed countries but in the developing countries of Africa, Asia and Latin America, leprosy is still considered a public health problem. Leprosy is a curable disease and early treatment will prevent further disabilities [1,2].

*Mycobacterium leprae *have an affinity for peripheral nerves. The nerve damage affects sensory, motor, and autonomic fibers. Sensory loss occurs earliest and is the most frequently affected modality but motor loss can also occur [3]. The most commonly involved nerves are the posterior tibial, ulnar, median, lateral popliteal and facial nerves [4]. Other nerves affected by the disease include the greater auricular, radial and the radial cutaneous nerves. Impaired sensation leads to trauma and secondary infection, which causes tissue damage and deformities. Loss of motor function produces disability. The end result is physical impairment.

The immunological response mounted by the host determines the clinical phenotype of the disease. Tuberculoid leprosy is the result of a high cell-mediated immune response. Granulomatous inflammation of the external fibrous sheath (epineuron) of peripheral nerves transforms it into a thick cover incapable of stretching under pressure resulting in nerve damage [5,6]. However, lepromatous leprosy is characterized by a humoral immune response. Lesions are intraneural and extra-fascicular with bacterial proliferation within the Schwann cells, leading to foamy degeneration and loss of the ability to regenerate [7].

Classification of the reactions is essential to determine therapy and predict prognosis [8]. Type 1 (reversal) reactions occur in tuberculoid disease presenting with acute inflammation. Acute neuritis results in impairment of nerve function, which if not treated rapidly, will lead to permanent damage causing peripheral sensory and motor neuropathy [9,10]. Type 2 reactions mainly affect lepromatous cases with multi-organ involvement [11]. The greater the infiltration of the skin and the higher the bacterial index (BI), the greater the risk of developing type 2 reactions [12].

The treatment of type 1 reactions is aimed at controlling the acute inflammation, easing pain and reversing nerve damage. Multi-drug therapy (MDT) should be continued during a reaction. Acute neuritis is treated early with oral corticosteroids to minimize nerve damage and thus prevent disability [13]. The majority of type 2 reactions require immunosuppression. The more severe cases require high doses of corticosteroids [14]. The recurrent nature of the condition means that steroid-induced side effects may become a significant problem.

Corticosteroids have potent anti-inflammatory and anti-proliferative actions [15]. Intralesional injection of corticosteroids has the advantage of achieving a high local concentration with no systemic side effects [16]. Our case presentation shows successful intraneural extra-fascicular corticosteroid injection in a patient with longstanding neuropathy secondary to leprosy resulting in nerve regeneration.

## Case presentation

A 60-year-old right-handed farmer from Upper Egypt was diagnosed with borderline leprosy in 1985. He finished a 2-year course of MDT (Dapsone, Clofazimine and Rifampicin) with improvement in his bacteriological index (BI). However, he was disabled secondary to right claw hand deformity from neglected nerve damage. He had no other medical or surgical problems. He had no known drug allergies and denied alcohol or tobacco consumption.

Neurological evaluation of the right hand revealed complete sensory loss on the palmar surface and 2/5 motor weakness in conjunction with claw deformity of all fingers. The right ulnar nerve was markedly thickened along its anatomical course. There was also mild tenderness of the right median nerve at the wrist level.

**Figure 1:**
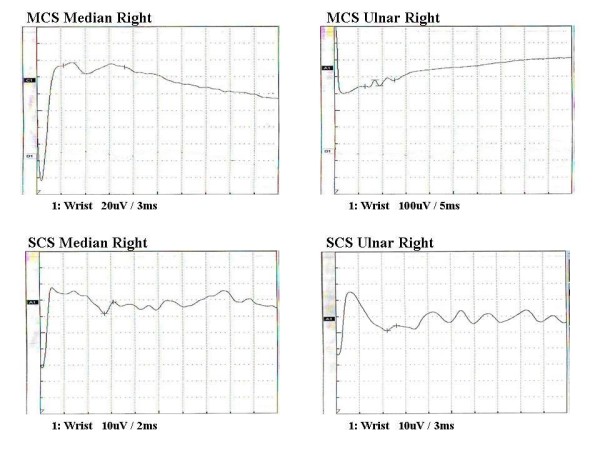
Nerve conduction velocity before corticosteroid injection.

Nerve conduction velocity testing (NCV) was performed on the right ulnar and median nerves. There was no conduction detected in the right ulnar nerve while the right median nerve showed a conduction velocity of 37 m/s. The sensory nerve conduction study for both nerves revealed complete absence of any sensory conduction (Figure 1).

After reviewing the risks and benefits, the patient consented to local corticosteroid injection of the right ulnar and median nerves. Monthly injection of 4 mg/ml dexamethasone phosphate was carried out for each nerve in the outpatient clinic for six consecutive months. After appropriate skin sterilization with alcohol pads, a 23-gauge needle was used to inject the ulnar nerve behind the right medial epicondyle and the median nerve at the palmer aspect of the right wrist as it enters the carpal tunnel. Premedication with a 20 mg intramuscular injection of belladonna extract was performed for prevention of vagal over-stimulation. There were no complications from the procedure. Serial monthly examination showed recovery of pain and deep pressure sensation over the hypothenar eminence that gradually extended to the fingers over the subsequent course of the therapy. Improvement of light touch sensation was delayed but eventually progressed in a similar fashion.

**Figure 2:**
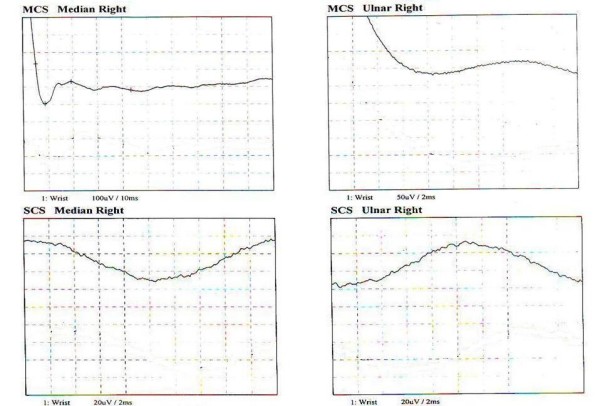
Nerve conduction velocity 6 months after corticosteroid injection.

NCV testing was repeated at the end of the treatment course. There was a marked improvement in the motor distal latency (DL) and motor conduction velocity (MCV) of the right median nerve (Figure 2). We were able to record the compound motor action potential (CMAP) in the right ulnar nerve. The sensory nerve action potential (SNAP) for the right ulnar and median nerves was clearly detected compared to results before the therapy.

## Discussion

We report the first case of successful nerve regeneration in neglected neuropathy secondary to leprosy after local injection of corticosteroids. Intraneuronal extra-fascicular injection of corticosteroids reverses the inflammatory and proliferative response to *Mycobacterium leprae*. This results in less mechanical pressure on the neuronal axons facilitating nerve regeneration and re-myelination. Our patient showed improvement in both the sensory and motor functions of those nerves. There were no reported complications or side effects from our approach. The advantage of local corticosteroid therapy is to deliver the medication at the site of action in a higher concentration compared to the oral route with minimal systemic side effects.

Our patient had right claw hand secondary to right ulnar and median nerve damage. He developed muscle weakness and contractures causing deformity and disability. Physical therapy with passive and active exercises was started to prevent fixation of joints. Muscle transplantation and tendon transfer may be appropriate in some cases as long as the joints remain mobile [1], however our patient refused any surgical intervention. Our treatment provided him with recovery of sensory and motor function of his right hand thus preventing further deformities.

Use of other therapies including oral thalidomide has been limited because of teratogenicity (phocomelia) and possible neurotoxicity. Clofazimine and pentoxifylline have both been used in type 2 reactions, but they are less effective than prednisolone or thalidomide [17,18]. Colchicine and chloroquine have also been used with limited effect. It remains to be seen whether tumor necrosis factor (TNF) blockade with biological agents will have a role to play in the management of type 2 reactions.

We are currently expanding our treatment protocol to present a series of similar cases. Eventually a randomized controlled study will test our hypothesis and compare it to other available treatment regimens.

## Conclusion

We report the first case of successful nerve regeneration in neglected neuropathy secondary to leprosy after local injection of corticosteroids. Intraneural extra-fascicular injection of corticosteroids improved the sensory and motor nerve function in our patient with borderline leprosy regardless of the duration of nerve function loss.

## Consent

Written informed consent was obtained from the patient for publication of this case report and any accompanying images. A copy of the written consent is available for review by the Editor-in-Chief of this journal.

## Competing interests

The authors declare that they have no competing interests.

## Authors' contributions

SN participated in the patient diagnosis and management, TR helped in patient management, ESAW participated in writing the manuscript, and AAA-E participated in patient management, follow-up and manuscript writing. All authors read and approved the final manuscript.
